# Comparative roles of *clpA* and *clpB* in the survival of *S*. Typhimurium under stress and virulence in poultry

**DOI:** 10.1038/s41598-018-22670-6

**Published:** 2018-03-14

**Authors:** Lal Sangpuii, Sunil Kumar Dixit, Manoj Kumawat, Shekhar Apoorva, Mukesh Kumar, Deepthi Kappala, Tapas Kumar Goswami, Manish Mahawar

**Affiliations:** 1 Indian Veterinary Research Institute, Division of Biochemistry, Izatnagar, 243122 U.P India; 2 Indian Veterinary Research Institute, Immunology Section, Izatnagar, 243122 U.P India

## Abstract

By assisting in the proteolysis, disaggregation and refolding of the aggregated proteins, Caseinolytic proteases (Clps) enhance the cellular survival under stress conditions. In the current study, comparative roles of two such Clps, ClpA (involved in proteolysis) and ClpB (involved in protein disaggregation and refolding) in the survival of *Salmonella* Typhimurium (*S*. Typhimurium) under different stresses and in virulence have been investigated. *clpA* and *clpB* gene deletion mutant strains (∆*clpA* and ∆*clpB*) of *S*. Typhimurium have been hypersensitive to 42 °C, HOCl and paraquat. However, the ∆*clpB* strain was comparatively much more susceptible (*p* < 0.001) to the above stresses than ∆*clpA* strain. ∆*clpB* strain also showed reduced survival (*p* < 0.001) in poultry macrophages. The hypersusceptibilities of ∆*clpB* strain to oxidants and macrophages were restored in plasmid based complemented (∆*clpB* + *pclpB*) strain. Further, the ∆*clpB* strain was defective for colonization in the poultry caecum and showed decreased dissemination to the spleen and liver. Our findings suggest that the role of ClpB is more important than the role of ClpA for the survival of *S*. Typhimurium under stress and colonization in chickens.

## Introduction

Food borne-infections account for about 86% of human cases of non-typhoidal salmonellosis^1^. *Salmonella enterica* serovar Typhimurium (*S*. Typhimurium) is one of the most frequently isolated serovars of *Salmonella* from food-borne infections^2^. *S*. Typhimurium causes mild to moderate gastroenteritis in healthy individuals^3^, however, it is associated with fatal infections in young, old and immunocompromised people^4,5^. In chickens, *S*. Typhimurium provokes very mild gastroenteritis. However, infected hens serve as chronic carriers without showing any clinical signs of infection but lay contaminated eggs^6^. Amongst the various food sources entailed in human *Salmonella* infection, poultry products have been reported to be the foremost complicit in outbreaks across the world (CDC, 2013)^7^. After entering via the oral route, *S*. Typhimurium reaches into the intestine of the host and penetrates epithelial linings. This is brought about with the aid of T3SS encoded by 40 kb region located in the *Salmonella* genome called *Salmonella* Pathogenecity Island (SPI) 1. The T3SS effector molecules stimulate the engulfment of bacteria into the epithelial membrane by mediating rearrangement of actin filaments and causing formation of membrane ruffles around bacteria^8^. Macrophages phagocytose the bacteria once *Salmonella* reaches sub-mucosa. T3SS2 effector molecules secreted by SPI2 helps in the survival and multiplication of *Salmonella* inside *Salmonella* containing vacuole (SCV) present within macrophages. Finally, localization occurs through blood circulation and disseminating macrophages into target organs of predilection especially lymph organs (spleen and caeca) and liver^9^. Different studies reveal differences in the extent of colonization of organs depending on the host infected. In poultry, major colonization is usually seen in caeca while spleen and liver harbour four orders of magnitude of bacteria lower than that of caeca^10^.

Inside the host *S*. Typhimurium encounters several stresses, including oxidants generated by the phagocytes and higher (42 °C) body temperature of poultry^11^. Superoxide anion (O_2_^−^), hydrogen peroxide (H_2_O_2_) and hypochlorous acid (HOCl) are some of the important reactive oxygen species (ROS) generated by phagocytic cells^12^. The exposure of *S*. Typhimurium to high temperature and oxidants results in unfolding and aggregation of proteins. These aggregates contain higher amounts of intermolecular β-sheet structures^13^ and are functionally inactive. The accumulation of protein aggregates hampers the cellular survival^14^ therefore they need to be taken care of at any cost.

To withstand such assaults, bacteria have evolved several mechanisms. These systems are composed of primary antioxidants, protein repair enzymes and various families of proteases and molecular chaperones. Proteases and molecular chaperones are categorized under heat shock proteins (Hsps) which specifically identify aggregated/misfolded proteins. Subsequently, Hsps cause either proteolysis or disaggregation and refolding^15,16^ of protein aggregates. Expression of molecular chaperones are found to be induced under stress conditions^17–19^.

In *S*. Typhimurium, three types of energy dependant proteases, including Clps family (*viz*. ClpA, ClpB, ClpX, ClpP), Lon and HslVU are present^20^. Clps are classified under the Hsp100 family proteins and function as both proteases and chaperones^21^. The ClpA and ClpB/Hsp104 chaperones belong to class 1 AAA + (ATPases associated with various cellular activities) protein superfamily^22,23^. ClpA is a part of the two-component protease (ClpAP) that binds protein substrates and presents them to the protease (ClpP) for degradation^24–27^. Recognition of substrate proteins is undertaken by the N- terminal domain of ClpA which also simultaneously unfolds the proteins^28,29^. This unfolding aids the proteolytic degradation occurring in the main proteolytic site of ClpP which receives these unfolded proteins via translocation through the hexameric body of ClpA^30,31^.

ClpB is an essential protein of heat shock response. Structurally, ClpB possesses a longer middle region known as ClpB/Hsp 104-linker which is essential for the chaperone activity^32,33^. Although sharing a 42% sequence identity and 64% sequence similarity with ClpA^34^, ClpB functions differently than ClpA. Instead of degradation, ClpB causes disaggregation and refolding of protein complexes in cooperation with DnaK, DnaJ and GrpE (KJE) chaperones^35^. ClpB first acts as a molecular chaperone and catalyses fragmentation of large protein aggregates into small fragments^36^. Subsequently, the activities of KJE resolubilise and refold these smaller fragments rendering their conformation more or less akin to their native forms^37^. Another thought suggests that ClpB/KJE act as a bichaperone in which the aggregated proteins are threaded into the ClpB/KJE complex and then unfolded proteins are extracted by the translocation activity of ClpB^38,39^.

ClpA and ClpB ATPases are implicated in stress tolerances of many organisms and are reported to protect vital cellular proteins during stress conditions^40–42^. Further, the ClpA and ClpB ATPases are found to play very important roles in the virulence of several bacterial pathogens^43–45^. However, the role of ClpA and the comparative importance of ClpA and ClpB in the stress survival and virulence of *S*. Typhimurium are not known. Here we evaluated and compared the roles of ClpA and ClpB in the survival of *S*. Typhimurium under stress and virulence in poultry. To accomplish this, we have generated *clpA* and *clpB* gene deletion mutants and complemented strains. Then their sensitivities to high temperature, oxidative stresses and intramacrophage survival were assessed. Further, we have investigated the effect of *clpA* and *clpB* gene deletions in the colonization of *S*. Typhimurium in chickens.

## Results

### Construction and confirmation of *clpA* and *clpB* gene deletion and complementation strains in *S*. Typhimurium

The PCR based analyses of *clpA* and *clpB* gene deletion mutants are shown in Supplementary Fig. S1, A and B. Test primers c located in the flanking regions of *clpA* gene amplified 296 bp in *∆clpA* and 2.4 kilobase pair (kb) in wild type (WT) strains, respectively (Supplementary Fig. S1A, *∆clpA* and WT lanes). Similarly, the test primers d designed in flanking regions of *clpB* gene amplified 566 base pair (bp) in *∆clpB* and 3 kb in WT strains respectively (Supplementary Fig. S1B, *∆clpB* and WT lanes).

Complementations of ∆*clpA* and ∆*clpB* strains were confirmed by RT-PCR. *clpA* specific primers j amplified 159 bp product in WT strain (Supplementary Fig. S2, lane WT). This amplicon was absent in ∆*clpA* strain (Supplementary Fig. S2, lane ∆*clpA*) and reappeared in ∆*clpA* + *pclpA* (complemented) strain (Supplementary Fig. S2, lane ∆*clpA* + *pclpA*). Similarly, by using *clpB* gene specific primers k, WT and complemented (∆*clpB* + *pclpB*) strains gave amplifications of 168 bp (Supplementary Fig. S2, WT and ∆*clpB* + *pclpB* lanes) which was absent in ∆*clpB* strain (Supplementary Fig. S2, ∆*clpB* lane).

**Figure 1:**
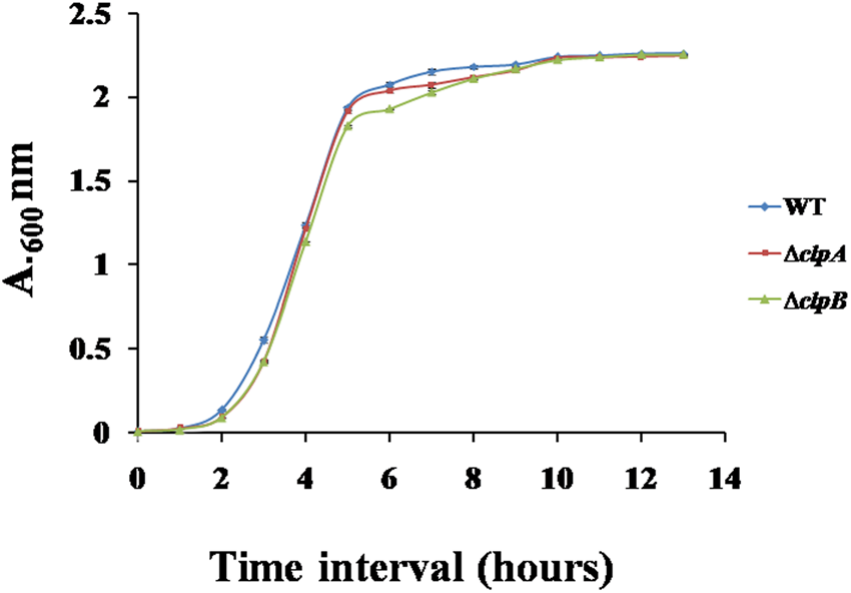
*In vitro* growth analysis of WT, ∆*clpA* and ∆*clpB* strains of *S*. Typhimurium. Isolated colonies of different strains were inoculated and grown in LB broth for overnight at 37 °C, 180 rpm. Overnight cultures were then diluted (at 1:100) in fresh medium. The optical densities were measured at 600 nm at an interval of one h. Experiment was performed two times. Data are presented as mean ± S.D. of three  replicates.

It is important to analyze the effect of gene deletion on *in vitro* growth of bacteria. Δ*clpA* and Δ*clpB* mutant strains did not exhibit any defective growth in LB broth (Fig. 1). Δ*clpA* and Δ*clpB* mutants and WT strains exhibited sigmoidal growth curves.

### Contribution of *clpA* and *clpB* in the survival of *S*. Typhimurium at 42 °C and oxidative stress

**Figure 2:**
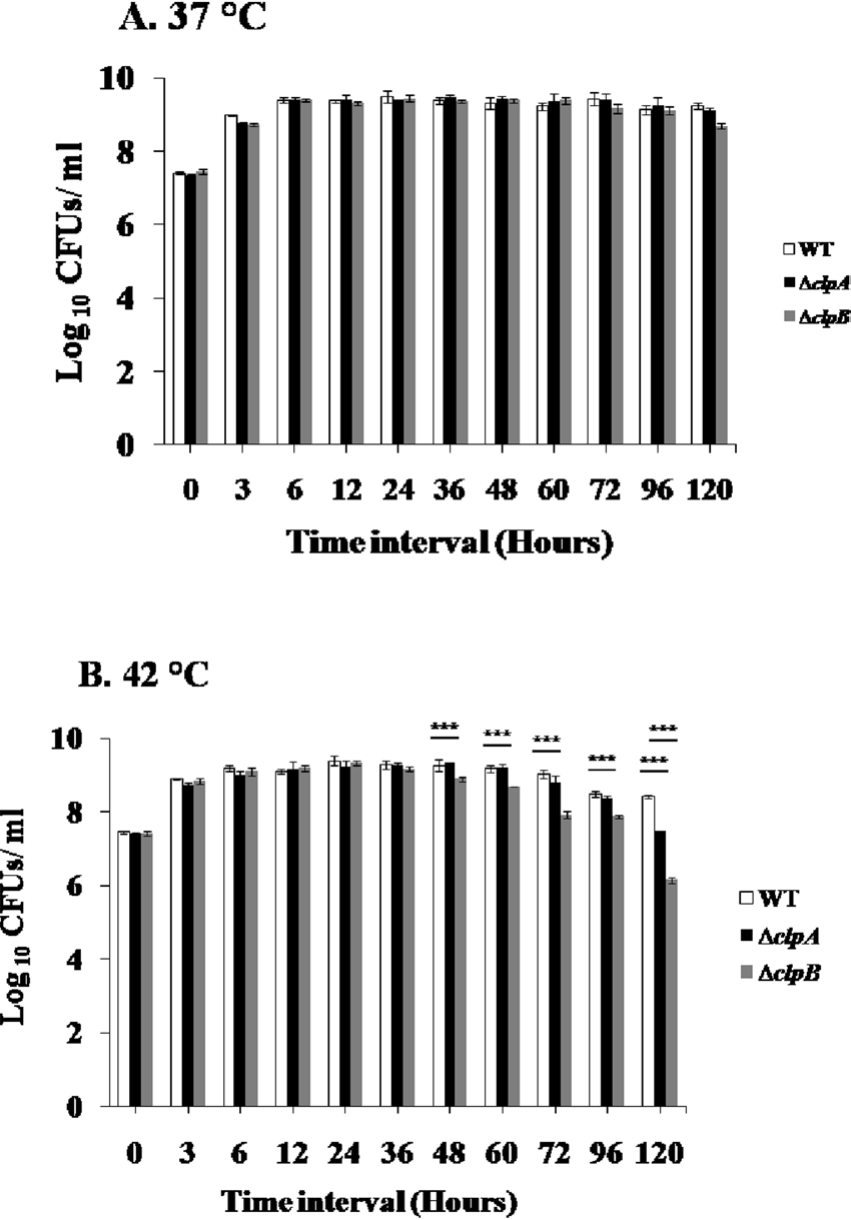
Growth of WT, ∆*clpA* and ∆*clpB* strains of *S*. Typhimurium at different temperatures. The overnight cultures of different strains were diluted in fresh media (1:100) and grown on shaker incubator either at 37 °C (**A**) or 42 °C (**B**). Aliquots were withdrawn at indicated time intervals, ten fold serially diluted and plated on HE agar plates. CFUs/ml were calculated following overnight incubation of plates. Data are presented as mean ± S.D. of three replicates (***denotes *p* < 0.001).

The body temperature of poultry is 42 °C, hence *S*. Typhimurium experience constant thermal stress inside birds^46^. Therefore, the contributions of *clpA* and *clpB* genes in the *in vitro* survival of *S*. Typhimurium at 42 °C have been evaluated. As compared to WT, ∆*clpA* and ∆*clpB* strains did not show any sensitivity to 37 °C (Fig. 2A). However, ∆*clpA* and ∆*clpB* strains were hypersusceptible (*p* < 0.001) to 42 °C exposure (Fig. 2B). In comparison to ∆*clpA* strain, ∆*clpB* strain showed hypersusceptibility to 42 °C (Fig. 2B). The numbers of bacteria recovered following 120 h of incubations [log_10_ colony forming unit(s) (CFUs)/ml as mean ± standard deviation (S.D.)] were 8.43 ± 0.032, 7.48 ± 0.008 and 6.15 ± 0.075 for WT, ∆*clpA* and ∆*clpB* strains respectively.

**Figure 3:**
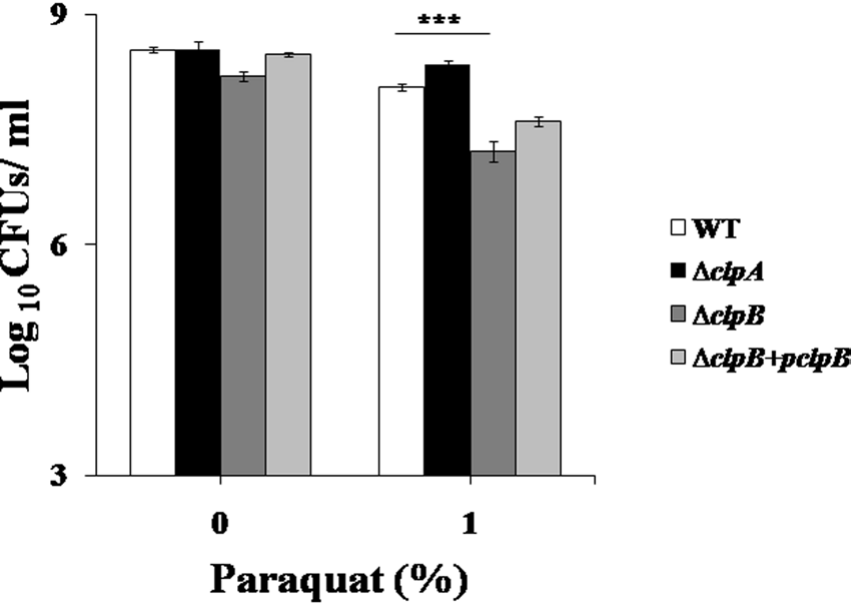
Sensitivities of WT, ∆*clpA* and ∆*clpB* strains of *S*. Typhimurium to paraquat. Mid-log grown cultures of WT, ∆*clpA*, ∆*clpB* mutants and ∆*clpA* + *pclpA* (complemented) strains of *S*. Typhimurium were exposed to 0 or 1% paraquat. Cultures were then ten fold serially diluted and plated on HE agar plates. CFUs/ml were calculated after overnight incubation of plates. Data are presented as mean ± S.D. of three replicates (***denotes *p* < 0.001).

Oxidative burst is an important part of the host immune response and proteins are primary targets of such responses. Next, susceptibilities of WT, ∆*clpA* and ∆*clpB* strains to various oxidants were evaluated. In comparison to ∆*clpA* and WT strains, ∆*clpB* strain was highly susceptible (*p* < 0.001) to paraquat (Fig. 3). Following two h of incubation with paraquat, the recovered viable numbers were (log_10_ CFUs/ml as mean ± S.D.) 8.05 ± 0.04, 8.35 ± 0.04 and 7.22 ± 0.12 in WT, ∆*clpA* and ∆*clpB* strains. Complemented (∆*clpB* + *pclpB*) strain exhibited intermediate susceptibility to paraquat with a recovery of 7.61 ± 0.06 (log_10_ CFUs/ml as mean ± S.D.) (Fig. 3).

Next, sensitivities of ∆*clpA* and ∆*clpB* strains to H_2_O_2_ were assessed_._ The exposure of ∆*clpA* and ∆*clpB* strains to 5 mM H_2_O_2_ did not show any hypersensitivity (*p* > 0.05) as compared to WT strain (Supplementary Fig. S3). Following exposure to H_2_O_2,_ we recovered (log_10_ CFUs/ml as mean ± S.D.) 9.26 ± 0.05, 9.15 ± 0.04 and 9.19 ± 0.16 viable bacteria in case of WT, ∆*clpA* and ∆*clpB* strains respectively.

**Figure 4:**
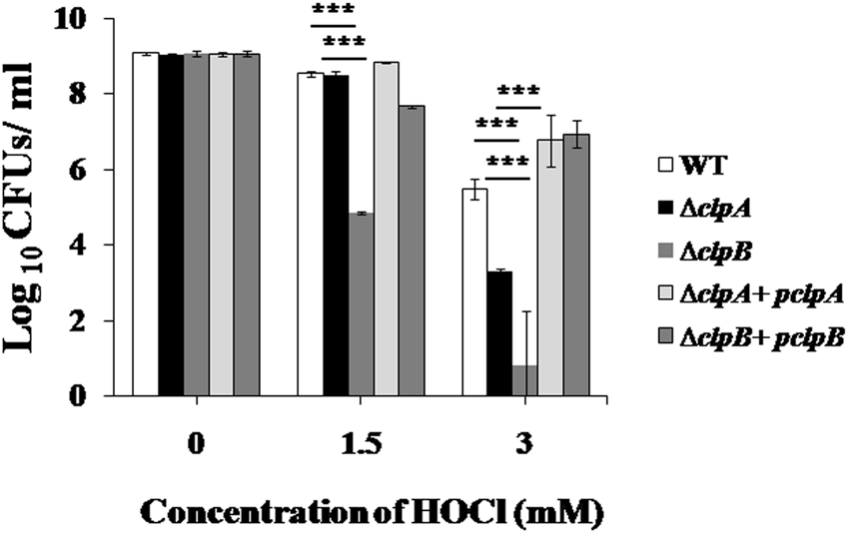
Sensitivities of ∆*clpA* and ∆*clpB* strains of *S*. Typhimurium to HOCl. Mid-log phase grown cultures of WT, ∆*clpA*, ∆*clpB* mutants and complemented (∆*clpA* + *pclpA* and ∆*clpB* + *pclpB*) strains of *S*. Typhimurium were exposed to 0, 1.5 and 3 mM HOCl for 2 h. Cultures were then serially diluted and plated on HE agar plates. CFUs/ml were enumerated after overnight incubation of plates. Data are presented as mean ± S.D. of three replicates (***denotes *p* < 0.001).

HOCl is one of the most potent oxidants generated by neutrophils and is a part of the oxidative burst encountered by *S*. Typhimurium upon internalization by phagocytic cells^47^. In comparison to WT strain, the ∆*clpB* strain was highly susceptible (*p* < 0.001) to 1.5 and 3 mM concentrations of HOCl. However, ∆*clpA* strain showed susceptibility (*p* < 0.001) to 3 mM but not to 1.5 mM HOCl (Fig. 4). Bacterial numbers recovered following 3 mM HOCl exposure (log_10_ CFUs/ml as mean ± S.D.) were 5.49 ± 0.27, 3.32 ± 0.04 and 0.82 ± 1.43 for WT, ∆*clpA* and ∆*clpB* strains respectively. Hypersusceptibilities of ∆*clpA* and ∆*clpB* strains to HOCl were restored in plasmid based complemented (∆*clpA* + *pclpA* and ∆*clpB* + *pclpB*) strains. Following incubation with 3 mM HOCl, the numbers of viable bacteria recovered (log_10_ CFUs/ml as mean ± S.D.) were 6.76 ± 0.68 and 6.94 ± 0.35 for ∆*clpA* + *pclpA* and ∆*clpB* + *pclpB* strains respectively.

### ∆*clpB* strain is highly susceptible to monocyte derived macrophages (MDM)

**Figure 5:**
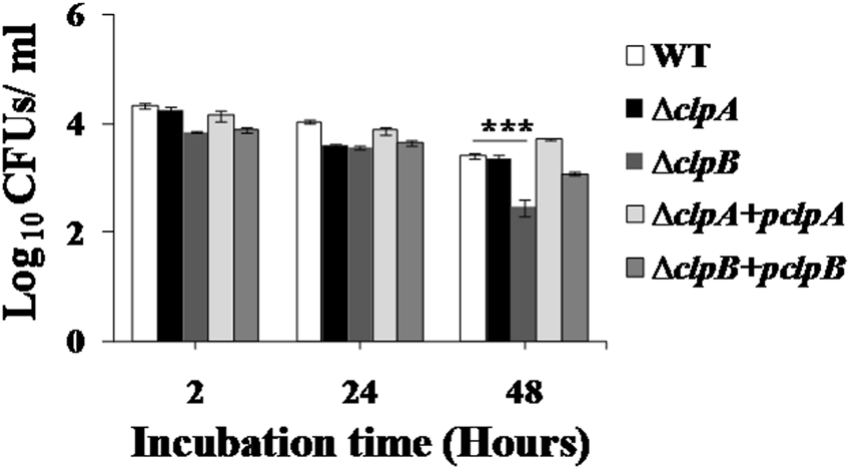
Sensitivities of WT, *∆clpA, ∆clpB, ∆clpA* + *pclpA* and *∆clpB* + *pclpB* strains to poultry macrophages stimulated by LPS. The mid log grown cultures were suspended in RPMI-1640. Macrophages were stimulated with LPS as described in materials and methods. The poultry macrophages were infected at MOI of 1:50 (macrophage: bacteria) for 2 h at 37 °C, 5% CO_2_. Macrophages were lysed by 0.1% TritonX-100. Bacteria were serially diluted and plated on HE agar plates and the number of bacteria recovered (log_10_ CFUs/ml as mean S.D.) were counted the next day. Data is representative of two individual experiments (n = 3) (***denotes *p* < 0.001).

The numbers of WT bacteria recovered (log_10_ as CFUs/ml as mean ± S.D.) following 24 and 48 h post infection were 4.037 ± 0.031 and 3.413 ± 0.043 respectively. The numbers of bacteria recovered from ∆*clpA* strain infected macrophages were 3.600 ± 0.048 and 3.355 ± 0.090 following 24 and 48 h of incubation. However, the ∆*clpB* strain showed defective intramacrophage survival (*p* < 0.001) as compared to WT and ∆*clpA* strains. We recovered 3.574 ± 0.040 and 2.460 ± 0.151 CFUs of ∆*clpB* mutant bacteria following 24 and 48 h of incubation. The function of *clpB* was partly restored in complemented (∆*clpB* + *pclpB*) strain which showed increased viability over *∆clpB* strain. Numbers of complemented strains recovered following 24 and 48 h of incubation were 3.654 ± 0.052 and 3.078 ± 0.036 respectively (Fig. 5).

### ∆*clpA* and ∆*clpB* strains accumulate more aggregated proteins

**Figure 6:**
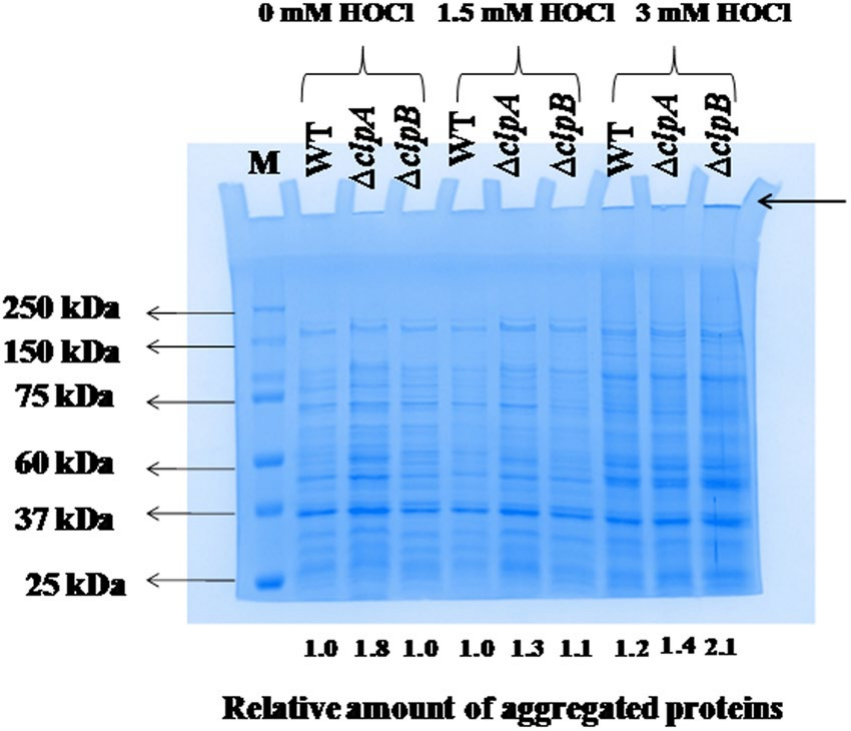
SDS-PAGE analysis of aggregated proteins in WT, ∆*clpA* and ∆*clpB* strains of *S*. Typhimurium. Mid-log grown cultures of various strains were exposed to different concentrations of HOCl for 30 min. Aggregated proteins from such cells were prepared and analysed on 10% SDS-gel. Relative quantification was done using ImageJ software(NIH) bundled with 32 bit Java 1.8.0 and expressed as fold difference from protein aggregates isolated from WT strain incubated with 0 mM HOCl. Quantification is indicated below each lane. Experiment was performed two times.

As ClpA and ClpB inhibit protein aggregations, we hypothesized higher levels of protein aggregates in ∆*clpA* and ∆*clpB* strains. Following incubations with PBS or 1.5 mM HOCl, we observed more amounts of protein aggregates in ∆*clpA* and ∆*clpB* strains as compared to WT counterpart. The amount of loading was normalized in terms of CFUs and was equivalent to 45 × 10^7^ CFUs/lane. Interestingly, the amount of aggregates was higher in ∆*clpA* strain as compared to ∆*clpB* strain (Fig. 6). Following 3 mM of HOCl exposure, we observed higher levels of aggregates in WT, ∆*clpA* and ∆*clpB* strains of *S*. Typhimurium. The amount of loading per lane was equivalent to aggregates isolated from 3 × 10^5^ CFUs. Some of these aggregates failed to enter in the gel (marked by arrow in the figure). WT strain did not show much increase in protein aggregates following HOCl treatment. We observed HOCl-dose dependent increase in amount of aggregates in ∆*clpB* strain. ∆*clpA* strain showed more amounts of aggregates in 0 and 1.5 mM HOCl treated samples than WT and ∆*clpB* strains.

### *clpB* contributes to the colonization in poultry caecum and dissemination of *S*. Typhimurium to spleen and liver

Our *in vitro* analyses suggest that *clpB* plays more important role than *clpA* in the survival of *S*. Typhimurium under heat and oxidative stresses (Figs 2, 3 and 4). This prompted us to compare the roles of *clpA* and *clpB* in the virulence of *S*. Typhimurium. *Salmonella* free birds were orally infected with WT or ∆*clpA* or ∆*clpB* strains of *S*. Typhimurium and their caecal colonizations were evaluated. We recovered *Salmonella* from caeca of all (4/4) chicks infected with WT strain of *S*. Typhimurium at all times post infection (Table 1). In birds infected with ∆*clpA* strain, 4/4 (100%) caeca were positive for *Salmonella* on 7 and 14 days post infection. After 21 days post infection, ∆*clpA* strain was recovered from 75% of the chicks (3 positive out of 4). However, ∆*clpB* strain was able to colonize initially in only 2 out of 4 (50%) chicks on 7 and 14 days post infection and eventually got cleared on 21 days (Table 1).

**Table 1:** Contribution of *clpA* and *clpB* in the caecal colonization of *S*. Typhimurium. Six days old chicks were orally inoculated with different strains of *S*. Typhimurium (as indicated in Table). At different times post infection, the birds were dissected and presence of *S*. Typhimurium and mutant strains were confirmed by PCR (as described in materials and methods).

Days post infection	positive/infected birds		
	WT (n = 4)	*∆clpA* (n = 4)	*∆clpB* (n = 4)
7	4/4 (100%)	4/4 (100%)	2/4 (50%)
14	4/4 (100%)	4/4 (100%)	2/4 (50%)
21	4/4 (100%)	3/4 (75%)	0/4 (0%)

**Figure 7:**
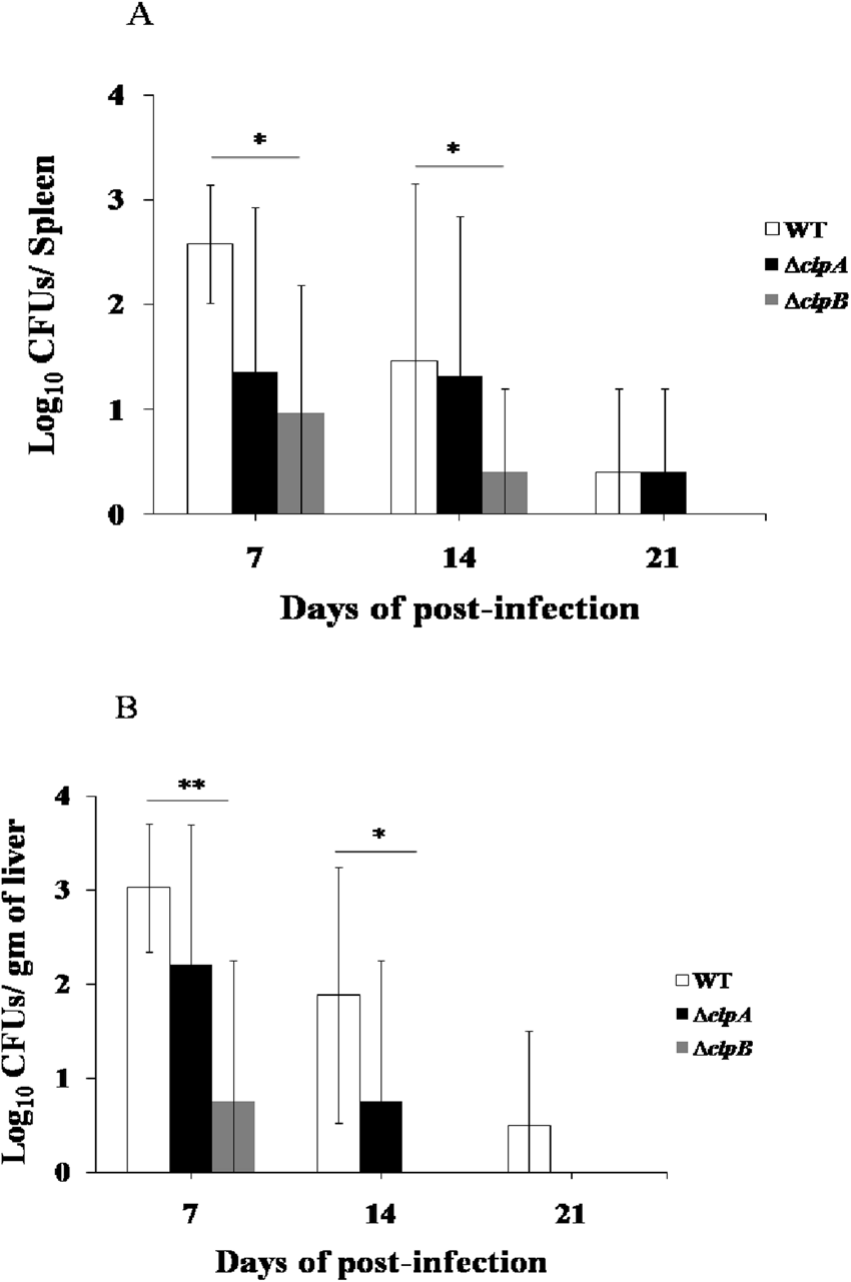
Bacterial burdens in the spleen (**A**) and liver (**B**) of *S*. Typhimurium, ∆*clpA* and ∆*clpB* infected birds. Half of the spleen and 100 mg of liver tissues were homogenized in PBS. One hundred microlitres of the homogenates were plated on HE agar plates. Colonies were counted following incubation of the plates. CFUs/spleen and CFUs/gm of liver were calculated. Data are presented as mean ± S.D. (n = 4) (**denotes *p* < 0.01, *denotes *p* < 0.05).

After concluding the role of *clpB* in the caecal colonization of *S*. Typhimurium, next, we analyzed the contribution of *clpB* in the dissemination of *S*. Typhimurium to poultry spleen and liver. We determined the bacterial loads in the spleen and liver on 7, 14 and 21 days post infection. In the spleen of WT strain infected birds, we obtained bacteria at all times post infection (Fig. 7A). The numbers of *Salmonella* recovered on 7, 14 and 21 days (log_10_ CFUs/spleen as mean ± S.D.) were 2.57 ± 0.56, 1.46 ± 1.69 and 0.400 ± 0.80 respectively. Similarly, bacteria were recovered on all times post infection from the spleen of ∆*clpA* strain infected chickens. The counts were (log_10_ CFUs/spleen as mean ± S.D.) 1.35 ± 1.57, 1.31 ± 1.52 and 0.40 ± 0.80 on 7, 14 and 21 days post infection (Fig. 7A). Interestingly as compared to that in WT strain infected birds, the bacterial loads in the spleen of ∆*clpB* strain inoculated chicks reduced significantly (*p* < 0.01) on 7 and 14 days post infection. On 21 days post infection, we did not recover any bacteria in the spleen of ∆*clpB* strain infected birds (Fig. 7A). The recovered numbers of bacteria (log_10_ CFUs/spleen as mean ± S.D.) on 7 and 14 days post infection were 0.96 ± 1.22 and 0.40 ± 0.80 respectively.

In liver, we recovered bacteria from WT strain infected birds at all times post infection (Fig. 7B). The numbers of WT bacteria recovered (log_10_ CFUs/gm of liver as mean ± S.D.) were 3.02 ± 0.68, 1.88 ± 1.36 and 0.5 ± 1.0 on 7, 14 and 21 days post infection respectively. In ∆*clpA* strain infected birds, we recovered 2.20 ± 1.49 and 0.75 ± 1.5 bacteria (log_10_ CFUs/gm of liver as mean ± S.D.) on 7 and 14 days post infection (Fig. 7B). However, the bacterial loads in the liver of ∆*clpB* strain infected birds were significantly (*p* < 0.01) less on 7 days post infection (0.75 ± 1.5). Following 14 and 21 days post infection; we did not recover any bacteria from the liver of ∆*clpB* strain infected birds (Fig. 7B).

## Discussion

*S*. Typhimurium encounters numerous stresses inside the host which primarily affect the integrity of cellular proteins. Molecular chaperones and proteases can refold or remove these abnormal proteins, thus play very important roles in maintaining the cellular homeostasis^48^. Clp proteases belong to AAA + class of proteins which are classified in to Class I and Class II types. Class I protease which include ClpA, ClpB and ClpC have two AAA + domains and degrade/disaggregate larger substrates while class II AAA + proteases like ClpX have only one AAA + domain and can only deal with smaller substrates. These proteins require various accessory proteins to execute their functions. ClpB complexes with DnaK, DnaJ and GrpE. ClpA, ClpX and ClpC associate with ClpP but each require a different set of adapter proteins. Like ClpS assists ClpA; MecA and YpbH coordinate with ClpC while ClpX complexes with SspB, RssB and UmuD^49^. Out of the known Clp proteases, we sought to analyse the relative importance of degradation versus disaggregation of protein aggregates in the survival of *S*. Typhimurium. In the current study, we have evaluated the comparative roles of ClpA (protein degradation chaperone) and ClpB (protein disaggregation chaperone) in the survival of *S*. Typhimurium under *in vitro* stress and in virulence.

First, we generated and confirmed *clpA* and *clpB* mutants and complemented (∆*clpA* + *pclpA* and ∆*clpB* + *pclpB*) strains (Supplementary Figs S1 and S2). ∆*clpA* and ∆*clpB* strains grew comparable to WT strain at 37 °C but were highly susceptible (*p* < 0.001) to 42 °C exposure (Fig. 2A and B). Following 120 h of exposure at 42 °C, (the log_10_ CFUs/ml; mean ± S.D. values for WT, ∆*clpA* and ∆*clpB* strain were 8.43 ± 0.032, 7.48 ± 0.008 and 6.15 ± 0.075 respectively), ∆*clpA* strain was more than 8 folds susceptible (*p* < 0.001) than WT strain (Fig. 2B). However, ∆*clpB* strain was more than 188 and 21 folds more susceptible (*p* < 0.001) than WT and ∆*clpA* strains respectively at 42 °C (Fig. 2B). Our experiments suggest that both ClpA and ClpB contribute to the survival of *S*. Typhimurium at 42 °C, however, ClpB plays a more crucial role in defending the temperature stress in this bacterium. Thomas and Baneyx observed defective recovery of *E. coli* ∆*clpB* mutant strain following 42 °C exposures^50^. ∆*clpA*^51^ or ∆*clpB*^52^ mutant strains in *B. suis* suffered temperature stress and showed reduced growth at 42 °C. Similarly, *clpB* is reported to play very important role in the adaptation or survival of *E. coli*, *H. pylori* and *Pseudomonas putida* to thermal stresses^41,53,54^. Interestingly, *clpA* and *clpB* genes get induced in *E*. coli, *S*. Typhimurium and *Myxococcus xanthus* following incubation at 42 °C^55–58^.

To eliminate the invading bacteria, phagocytes generate a battery of ROS including O_2_^−^, H_2_O_2_, highly toxic hydroxyl radicals and HOCl. Paraquat (methyl viologen) is a superoxide generating compound^59^. ∆*clpB* strain was about 8 folds more susceptible (*p* < 0.001) than WT strain to paraquat (recovered viable numbers were 7.22 ± 0.12 and 8.05 ± 0.04 for ∆*clpB* and WT strains, respectively; Fig. 3). Complemented ∆*clpB* strain (∆*clpB* + *pclpB* strain) showed intermediate sensitivity to paraquat (Fig. 3). Next, we evaluated the susceptibilities of ∆*clpA* and ∆*clpB* strains to H_2_O_2_. ∆*clpA* and ∆*clpB* strains did not show hypersusceptibility (*p* > 0.05) to H_2_O_2_ (Supplementary Fig. S3). HOCl is reported to be 100-folds more toxic than H_2_O_2_^60^. We next analyzed the sensitivities of WT, ∆*clpA* and ∆*clpB* strains to HOCl. ∆*clpB* strain was much more susceptible to HOCl than ∆*clpA* strain. At 3 mM HOCl concentration, ∆*clpA* strain was 173 folds more susceptible (*p* < 0.001) than WT strain (Fig. 4). The susceptibility of ∆*clpB* strain (in comparison to WT strain) was more than 4792 folds (*p* < 0.001) at 1.5 mM and 3633 folds (*p* < 0.001) at 3 mM HOCl (following 1.5 mM HOCl treatment viable numbers for WT and ∆*clpB* were 8.53 ± 0.06 and 4.85 ± 0.03. While after 3 mM HOCl exposure, recovered viable numbers for WT and ∆*clpB* (log_10_ CFUs/ml; mean ± S.D.) were 5.49 ± 0.27 and 0.82 ± 1.43 respectively). Interestingly in comparison to ∆*clpA* strain, ∆*clpB* strain was highly susceptible to HOCl (4423 folds at 1.5 mM and 21 folds at 3 mM HOCl). The recovered CFUs/ml following incubation of ∆*clpA* at 1.5 and 3 mM HOCl were 8.49 ± 0.11 and 3.32 ± 0.04 respectively. Taken together, our *in vitro* experiments suggest that *clpB* is more important than *clpA* in the survival of *S*. Typhimurium under superoxides and HOCl induced oxidative stress.

Lourdault *et al*. reported the high susceptibility of *clpB* mutant strain of *L. interrogans* to butyl peroxide^61^. However, *clpA* and *clpB* gene deletion in *B. suis* did not affect the sensitivity to H_2_O_2_^52^. Conversely, upregulated expression of *Ehrlichia chaffeensis clpB* gene was observed following infection of macrophages^62^, suggesting an important role of ClpB protein under oxidative stress. *S*. Typhimurium encodes three catalases, including KatE, KatG and KatN^63^ which catalytically degrades H_2_O_2_. Catalases might be active at similar levels in all these three strains which might be the reason we did not observe hypersusceptibility of ∆*clpA* and ∆*clpB* strains to H_2_O_2_.

LPS stimulated macrophages generate robust immune response^64^. After 24 h of incubation, the ∆*clpA* and ∆*clpB* strains were about 3 folds more susceptible in macrophages than WT strain of *S*. Typhimurium. Following 48 h of incubation, ∆*clpB* strain was about nine and eight folds more susceptible in macrophages as compared to WT and ∆*clpA* strains respectively (Fig. 5). Similarly, defective intramacrophage survival of ∆*clpB* strains of *Franciscella* and *Coxiella burnetii* have been observed^65–68^. However, *clpA* gene was found to be dispensable for intramacrophage growth of *Brucella suis*^51^. Further, upregulation of *P. salmonis* and *Ehrlichia chaffeensis clpB* has been observed following incubation of these organisms with SHK-1 cell lines and macrophages^62,69^. These data suggest a crucial role of *clpB* in the intramacrophage survival of bacterial pathogens.

Our SDS- gel analysis revealed greater amounts of protein aggregates in ∆*clpA* and ∆*clpB* strains than that in WT strain of *S*. Typhimurium. Interestingly, we observed higher amounts of protein aggregates in ∆*clpA* strain than that in ∆*clpB* strain (Fig. 6). Following incubation of *clpB* gene deletion strain of *E. coli* at 42 °C, increased aggregation of pre S2-β-galactosidase was observed^70^. In a separate study, degradation of green fluorescent protein aggregates at 42 °C was observed in ∆*clpB* and WT strains but not in ∆*clpA* strain of *Brucella suis*^52^. ClpA might be actively involved in degrading the protein aggregates in ∆*clpB* strain that could be the reason we did not observe higher levels of aggregates in ∆*clpB* strain as compared to ∆*clpA* strain.

Following exposure of 3 mM HOCl we have observed more protein aggregates in WT, ∆*clpA* and ∆*clpB* strains of *S*. Typhimurium. Some of these aggregates were resistant to SDS and beta-mercaptoethanol (β-ME) and failed to enter in stacking gel (Fig. 6, marked by arrow). Cell has a limited capacity to refold/remove aggregated proteins. The protein aggregates formed under severe stress (3 mM HOCl in our experiment) might be beyond the repair/removal capabilities of cellular chaperone/protease systems^71^. Further, we have observed significant amount of killing of WT as well as of mutant strains following exposure to 3 mM HOCl (Fig. 4).

*S*. Typhimurium primarily colonizes in the caecum of young chicks^72^ and disseminates to spleen and liver. Eventually these birds serve as a carrier and lay contaminated eggs. As compared to WT and ∆*clpA* strains, ∆*clpB* strain was highly defective in caecal colonization and dissemination to spleen and liver (Table 1 and Fig. 7). Similarly, *clpA* deletion did not affect the colonization of *B. suis* and *H. pylori* in mice^51,73^. However, following inoculation of a pool of transposon mutants in chickens, defective recovery of a *clpB* gene insertion mutant was observed^74^. Further, in a competitive experiment, where pools of WT and *clpB* mutant of *S*. Typhimurium were inoculated in to chickens, *clpB* gene deletion strain showed defective fitness^75^. *clpB* gene deletion strains of *Listeria monocytogenes*^21^, *Francisella tularensis*^66^ and *Leptospira interrogans*^61^ exhibited attenuated virulence and defective survival in animal models. In brief, our data and previous reports suggest that *clpB* is more important than *clpA* for the survival of bacterial pathogens in the host.

Both ClpA and ClpB play important roles in preventing protein aggregations in the cell. As ClpA degrades protein aggregates, protein pool in the cell needs to be replenished via translational synthesis. While ClpB is involved in disaggregation and refolding of existing protein aggregates which would be a rapid and energy efficient way for restoration of protein function(s) in the cell (Supplementary Fig. S4). Therefore, ClpB would play more important role than ClpA in the cellular survival under stress conditions. Consistence to this hypothesis, in our experiments we observed that *clpB* gene deletion strain of *S*. Typhimurium was much more susceptible to different stresses *in vitro* (than *∆clpA* strain) and showed defective virulence in chickens.

## Methods

### Ethical Statement

All animal experiments were approved by the Institutional Animal Ethics Committee (IAEC), Indian Council of Agricultural Research-Indian Veterinary Research Institute (ICAR-IVRI), Izatnagar, India with the approval file No. F.26-1/2015–2016/J.D.(R). All animal experimentations were performed in accordance with the guidelines and regulations of IAEC, ICAR-IVRI, Izatnagar, India.

### Bacterial strains and plasmids

*S*. Typhimurium E-5591 was obtained from National *Salmonella* Centre (Veterinary type), Division of Bacteriology and Mycology, Indian Veterinary Research Institute (IVRI), Izatnagar, India. The NEBα strain of *E. coli* was obtained from New England BioLabs. The plasmid pQE60 was procured from Qiagen, Hilden, Germany. The plasmids pKD4, pKD46 and pCP20 were a kind gift from Dr. Robert J. Maier, Department of Microbiology, University of Georgia, Athens, GA, USA.

### Culturing of *S*. Typhimurium

*S*. Typhimurium, its isogenic mutants and complemented strains were grown in Luria Bertani (LB) broth or Hektoen Enteric (HE) agar as described earlier^76^. Ampicillin at the concentration of 100 µg/ml was included while culturing the complemented strain.

### Construction of *clpA* and *clpB* gene deletion mutants and complemented strains

Primers utilized in this study are listed in Table 2. The *clpA* and *clpB* gene deletion mutants and complemented strains in *S*. Typhimurium were constructed as described earlier^76^. Briefly, the FRT flanked kanamycin cassettes were amplified using pKD4 plasmid as template. The kanamycin cassettes were electroporated in lambda red recombinase expressing *S*. Typhimurium. Positive recombinants were selected on kanamycin plates and confirmed by PCR. The kanamycin cassettes were then removed by flp recombinase. The *clpA* and *clpB* deletions mutants were confirmed by c and d primers located about 200 bp upstream and downstream from *clpA* and *clpB* genes. The gene deletion mutant strains were designated as ∆*clpA* and ∆*clpB*.

**Table 2:** List of primers used in this study.

Sl. No.	Name of primer	Sequence	Specific Purpose
a.	UP*clpA* Forward	5′ AAAAGCCTGAATGCAGGTATAAAAATTGGGGGAGGTGCCTTGTGTGTAGGCTGGAGCTGCCT 3′	To amplify kanamycin cassette with flanking regions of *clpA*
	UP*clpA* Reverse	5′ CGGGCGCTAAGGCCCGGTTTGTACGACAGTGAAACGAAGACATATGAATATCCTCCTTA 3′	
b.	UP*clpB* Forward	5′ TAATCTCCAGTAGCAATTTTGACCTGTTATGGGAGGAGTTTTGTGTAGGCTGGAGCTGCTTC 3′	To amplify kanamycin cassette with flanking regions of *clpB*
	UP*clpB* Reverse	5′ AAACGAGCCCGTCAGGGCCCGTTTTATTCAAATTTGTGACCATATGAATATCCTCCTTA 3′	
c.	*clpA* test deletion Forward	5′ AGAACGTGCAACGCAATTGATG 3′	To confirm the deletion of *clpA* from *S*. Typhimurium
	*clpA* test deletion Reverse	5′ GGTTTGTACGACAGTGAAACGA 3′	
d.	*clpB* test deletion Forward	5′ CTGGCGAATACCGGCGTT 3′	To confirm the deletion of *clpB* from *S*. Typhimurium
	*clpB* test deletion Reverse	5′ ACAGACTTCTTAACGAAGCTTT 3′	
e.	*clpA* amplification Forward	5′ ATATATGGATCCATGCTCAATCAAGAACTGGAAC 3′	To amplify and clone *clpA* in pQE60 (for complementation)
	*clpA* amplification Reverse	5′ ATATATAAGCTTTTAGTGCGCGGCTTCCGG 3′	
f.	*clpB*-pQE60_XhoI Forward	5′-ATATATCTCGAGATGCGTCTGGATCGTCTTAC-3′	To amplify and clone *clpB* in pQE60 (for complementation)
	*clpB*-pQE60_BamHI Reverse	5′ATATATGGATCCTTACTGCACTGCCACAATAC-3′	
g.	pQE60-*clpB* Forward	5′ATAAACAAATAGGGGTTCCGCG 3′	To confirm *clpA-*pQE60 clone
	pQE60-*clpB* Reverse	5′ GGCGGCAACCGAGCGTTCT 3′	
h.	pQE60-*clpB* Forward	5′ATAAACAAATAGGGGTTCCGCG 3′	To confirm *clpB-*pQE60 clone
	pQE60-*clpB* Reverse	5′ GGCGGCAACCGAGCGTTCT 3′	
i.	*typh* Forward	5′ TTGTTCACTTTTTACCCCTGAA 3′	*S*. Typhimurium specific primer. For confirmation of *S*. Typhimurium by amplifying *typh* gene
	*typh* Reverse	5′ CCCTGACAGCCGTTAGATATT 3′	
j.	*clpA* RT-PCR Forward	5′ ATGGCGCGTGTGATTCAGGAT 3′	To confirm expression of *clpA* by RT-PCR
	*clpA* RT-PCR Reverse	5′ GGCTTCCGGCTTGTGCTTTTGC 3′	
k.	*clpB* RT-PCR Forward	5′ CGGTTCCGATCTCATTCAGG 3′	To confirm expression of *clpB* by RT-PCR
	*clpB* RT-PCR Reverse	5′ TGAGCAATAGAAGCGATGTGTT 3′	

For complementations, the *clpA* and *clpB* genes were amplified by primers e and f and cloned into plasmid pQE60 (*clpA* at HindIII and BamHI restriction sites and *clpB* at XhoI and BamHI restriction sites). The positive recombinant plasmids were introduced into the Δ*clpA* and Δ*clpB* strains by electroporation. The positive colonies were confirmed by PCR using g and h primers (Table 2). The complemented strains were designated as ∆*clpA* + *pclpA* and ∆*clpB* + *pclpB*.

### Confirmation of transcription in complemented strains by Reverse Transcriptase (RT) – Polymerase chain reaction (PCR)

Overnight grown cultures of WT, ∆*clpA*, ∆*clpB*, ∆*clpA* + *pclpA* and ∆*clpB* + *pclpB* strains of *S*. Typhimurium were harvested by centrifugation. RNA from such pellets were isolated using Trizol reagent. RNA samples were treated with RNase-free DNase I and then dissolved in nuclease free water. All RNA samples were tested for contamination of DNA using *S*. Typhimurium *clpA* and *clpB* gene specific primers (Table 2 j and k).

RT- PCR was performed according to the protocol as described in Superscript VILO cDNA synthesis kit (Invitrogen). In brief, in 20 µl reactions, 0.5 µg of RNA samples were mixed with 4 µl of 5 × VILO reaction mix and 2 µl of 10 × Superscript enzyme mix. cDNA was synthesized by incubation of the above mix at 25 °C for 10 minutes (min) followed by 42 °C for 60 min and termination at 85 °C for 5 min. Part of *clpA* and *clpB* genes were PCR amplified using these cDNA samples as templates and j and k primers (Table 2).

### Growth curve study

The growth of WT, Δ*clpA* and Δ*clpB* strains was analyzed as described earlier^77^. In brief, isolated colonies of WT, Δ*clpA* and Δ*clpB* strains were grown overnight in LB broth. Overnight cultures were diluted (1: 100) in fresh LB broth and grown in a shaker incubator at 180 revolutions per minute (rpm), 37 °C. Aliquots were withdrawn at one h of intervals and optical densities (O.D.) were measured at 600 nanometre (O. D._600_ nm).

### Susceptibilities of Δ*clpA* and Δ*clpB* strains to 42 °C

Overnight cultures of WT, Δ*clpA* and Δ*clpB* strains were diluted 100 folds in fresh LB broth. The cultures were then incubated either at 37 °C or at 42 °C. Aliquots were withdrawn at 0, 3, 6, 12, 24, 36, 48, 60, 72, 96 and 120 h post incubation, serially diluted with 1 × phosphate buffered saline (PBS) and plated on HE agar plates. The plates were incubated at 37 °C for overnight. CFUs/ml were then calculated.

### Evaluation of *in vitro* susceptibilities of Δ*clpA* and Δ*clpB* mutant strains to different oxidants

Overnight cultures of different strains were sub-cultured in fresh LB broth (at the ratio of 1:100) and incubated in a shaking incubator at 37 °C. The mid log phase grown cultures were then exposed to different concentrations of paraquat, H_2_O_2_ and HOCl (sodium hypochlorite, NaOCl, Sigma) for 2 h. Aliquots were withdrawn, serially diluted and plated on HE agar plates. CFUs/ml were calculated following incubation of plates at 37 °C for overnight.

### Susceptibility of WT, Δ*clpA* and Δ*clpB* strains to macrophages

The susceptibilities of WT, Δ*clpA*, Δ*clpB* and complemented (∆*clpA* + *pclpA* and ∆*clpB* + *pclpB*) strains of *S*. Typhimurium to monocyte derived macrophages (MDM) were determined as described earlier^78^ with minor modifications. Briefly, heparinised poultry blood was layered over equal amount of Histopaque-1077 (Sigma) and mononuclear cells (MNCs) were recovered by centrifugation at 1300 × *g* for 30 min at 25 °C. MNCs were washed two times with RPMI-1640 media (HiMedia) supplemented with 2% chicken serum, 8% fetal bovine serum and 1 × antibiotic/antimycotic solution (Gibco). The cells were counted by trypan blue dye exclusion method. MNCs were adjusted to 2 × 10^6^ cells/ml in similar media. Then, the cells were seeded in 24 well cell culture plates at the number of 1 × 10^6^ cells/well and incubated for 6 h at 37 °C/5% CO_2_. Non-adhered cells were removed by washing. Cells were stimulated with *Salmonella enterica serovar* Typhimurium LPS (Sigma) at 0.5 µg/ml for 48 h. Following incubation, the cells were washed with antibiotic free media and infected with WT, Δ*clpA*, Δ*clpB* or complemented strains of *S*. Typhimurium at multiplicity of infection (MOI) of 1:50 (macrophages:bacteria). The infection was simulated by centrifugation at 120 × *g* for 10 min at 25 °C. The cell- bacterial mix was incubated for 2 h. To kill non invaded bacteria the mix was incubated in gentamicin (50 μg/ml) containing media for 90 min. The cells were then incubated in gentamicin (10 μg/ml) containing media. Following 24 or 48 h of incubation, the cells were lysed with 0.1% Triton X-100 and lysates were 10 folds serially diluted and plated on HE agar plates. Plates were incubated at 37 °C and colony forming units (CFUs)/ml were determined.

### Analysis of protein aggregations

Overnight grown cultures of WT, Δ*clpA* and Δ*clpB* strains were diluted in fresh LB broth and incubated at 37 °C and 180 rpm for 3 h. The cultures were then exposed to 0, 1.5 and 3 mM concentrations (final) of HOCl for 30 min. Bacteria were harvested by centrifugation at 7000 rpm for 10 min. Protein aggregates from such exposed cultures were isolated as described earlier^79^. In brief, the bacterial pellets were suspended in 500 µl of 50 mM Tris(hydroxymethyl) aminomethane (Tris) (pH 7.4), 5 mM ethylenediaminetetraacetate (EDTA), 20% sucrose and 1 mg/ml lysozyme. The mixtures were incubated at 25 °C for 10 min and diluted by addition of 5 volumes of 30 mM Tris buffer (pH 7.4). Following 20 seconds of brief sonication, mixtures were supplemented with 10 mM magnesium chloride (MgCl_2_) and DNaseI. Unbroken cells were removed by centrifugation at 2,000 × *g* for 2 min. Supernatants were incubated with 0.5% Triton X- 100 for 15 min at 25 °C. The insoluble cell fractions (protein aggregates) were recovered by centrifugation at 8,000 × *g* for 15 min at 25 °C. The protein aggregates were analyzed by sodium dodecyl sulphate-polyacrylamide gel electrophoresis (SDS-PAGE) under reducing conditions. Loading was normalized in terms of CFUs/ml. Relative amounts of protein aggregates in different treatments/groups were analysed by ImageJ software (NIH) bundled with 32 bit Java 1.8.0. Sum of peak area in different lanes were calculated using the software. Total sum of peak area in 0 mM HOCl treated WT sample (lane) is considered as one.

### Analysis of virulence in poultry

One day old chicks were procured from ICAR-Central Avian Research Institute (CARI), Izatnagar, India and provided with *adlibitum* feed and water. The birds were screened for the presence of *Salmonella* spp. as described earlier^76^. The *Salmonella* free birds were divided into three groups. At the age of six days (~1 week) they were orally infected with WT or Δ*clpA* or Δ*clpB* strains at a dose of 1 × 10^9^ CFUs/bird. Following 7, 14, and 21 days post-infection, 4 birds were sacrificed from each group. Caecal colonization and bacterial burdens in liver and spleen were assessed as described elsewhere^76^.

### Statistical analysis

Data were analyzed by SPSS. Comparisons between multiple groups were done by using one way analysis of variance (ANOVA) followed by Post - hoc Tukey alpha test. *p* < 0.05 was considered significant among different test groups.

### Data availability

The data analysed and generated during the current study are available from the corresponding author.

## Electronic supplementary material

Supplementary information
