# Supplementary material for: Comparative roles of clpA and clpB in the survival of S. Typhimurium under stress and virulence in poultry
Source: Sci Rep. 2018 Mar 14;8:4481. doi: 10.1038/s41598-018-22670-6 (PMC5852057; doi:10.1038/s41598-018-22670-6)

Supplementary Information

Comparative roles of *clpA* and *clpB* in the survival of *S*. Typhimurium under stress and virulence in poultry

Lal Sangpuii[1,+], Sunil Kumar Dixit[2,+], Manoj Kumawat[1], Shekhar Apoorva[1], Mukesh Kumar[1], Deepthi Kappala[2],Tapas Kumar Goswami[2], and Manish Mahawar[1,*]

[1]Division of Biochemistry, Indian Veterinary Research Institute, Izatnagar, 243122, U.P, India
[2]Immunology Section Indian Veterinary Research Institute, Izatnagar, 243122, U.P, India
*corresponding author: Manish Mahawar, Ph.D, Senior Scientist, Animal Biochemistry, Indian Veterinary Research Institute, Izatnagar, 243122, U.P, India, manishbiochemistry@gmail.com

Supplementary Figures

Supplementary Fig. S1. 1 % Agarose gel analysis of construction and confirmation of *clpA* (Fig. *A*) and *clpB* (Fig. *B*) gene deletion mutants in *S*. Typhimurium. Kanamycin cassettes were amplified and fused with flanking regions of *clpA* and *clpB* genes and transformed to *S*. Typhimurium. The positive recombinants were selected on kanamycin plates and confirmed by PCR. The kanamycin cassettes were removed by flp recombinase. Mutants were confirmed by PCR. The image displayed is cropped from the full-length gels. The full-length gels are presented in Supplementary Figure S1 A and B.

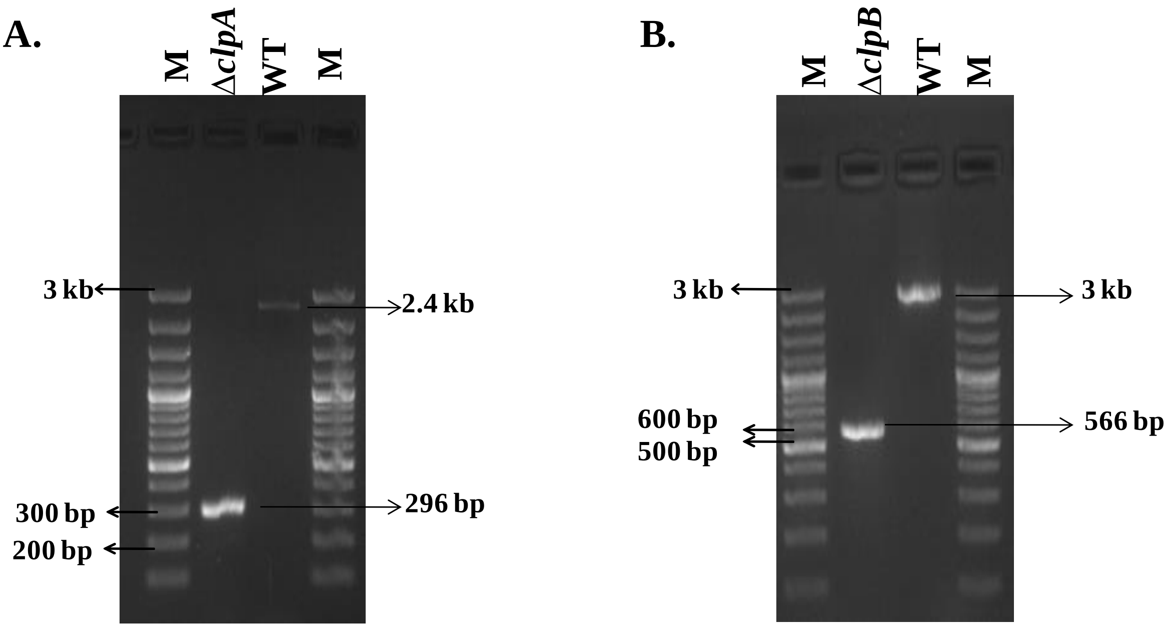

Supplementary Fig. S2. RT-PCR analysis of ∆*clpA* and ∆*clpB* mutants and complemented strains. RNA was isolated from WT, ∆*clpA* and ∆*clpB* mutants; and ∆*clpA*+ *pclpA* and ∆*clpB*+ *pclpB* complemented strains. cDNA were synthesized from these RNA samples. *clpA* and *clpB* genes were amplified from cDNA using *clpA* or *clpB* specific primers and analysed on 1.5 % agarose gel. The samples loaded in lanes are depicted in figure. M is 100 bp DNA ladder.

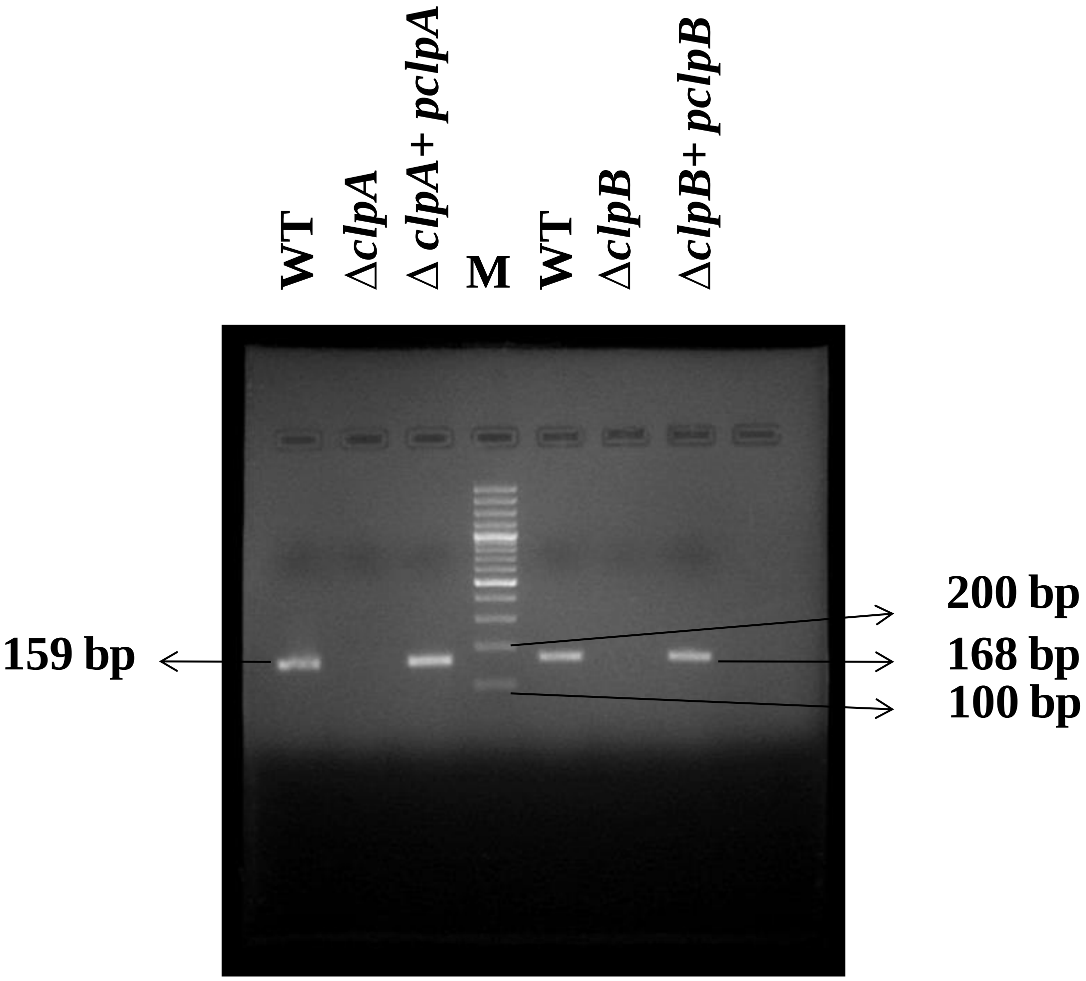

Supplementary Fig. S3. Sensitivities of Δ*clpA* and Δ*clpB* strains of *S*. Typhimurium to H_2O_2. The mid-log grown cultures of WT, Δ*clpA* and Δ*clpB* strains were exposed to 0 or 5 mM H_2O_2 for 2 hours. The cultures were then serially diluted and plated on HE agar plates. The differential sensitivities were estimated by enumerating the colonies. Data are presented as mean ± S. D. (n=3).

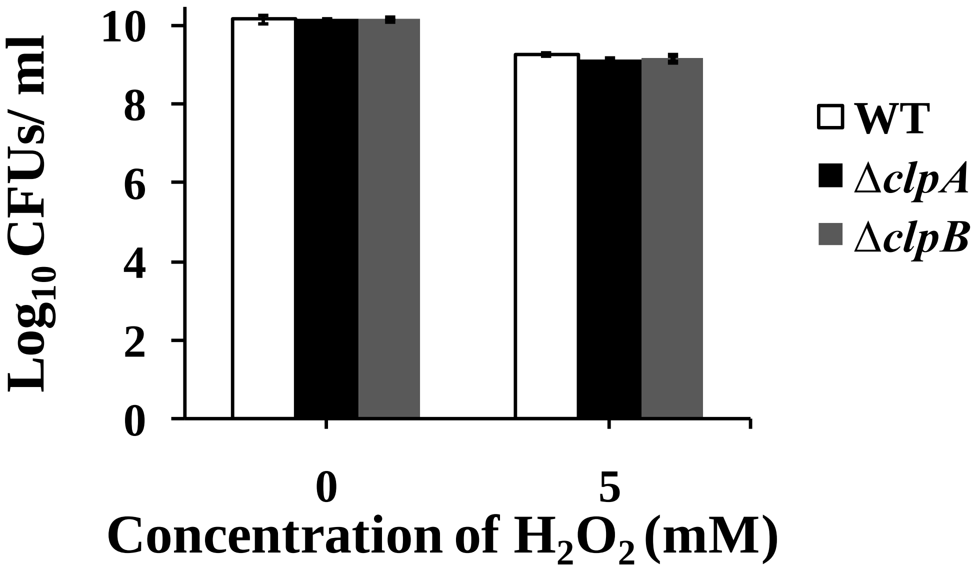

Supplementary Fig. S4. Proposed model. Temperature and oxidative stress cause unfolding of native proteins which results in the exposure of their hydrophobic residues and subsequent aggregation. Aggregated proteins can have two fates: either undergo degradation by the combine activity of ClpAP and protein pool in the cell will be replenished via ribosomal translational synthesis, or undergo disaggregation by ClpB followed by refolding by DnaKJE back to their native form. Hydrophobic residues in unfolded proteins are represented by black dots.

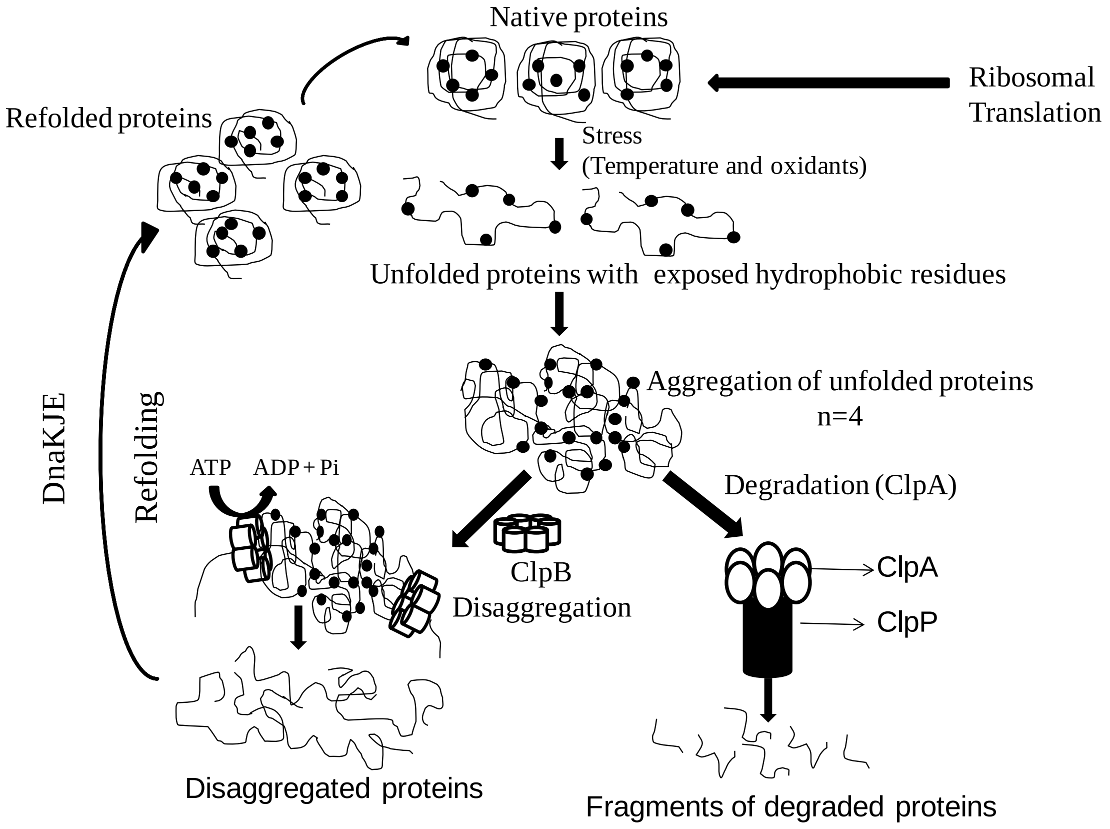

Full length gel of Supplementary Fig. S1 (A)

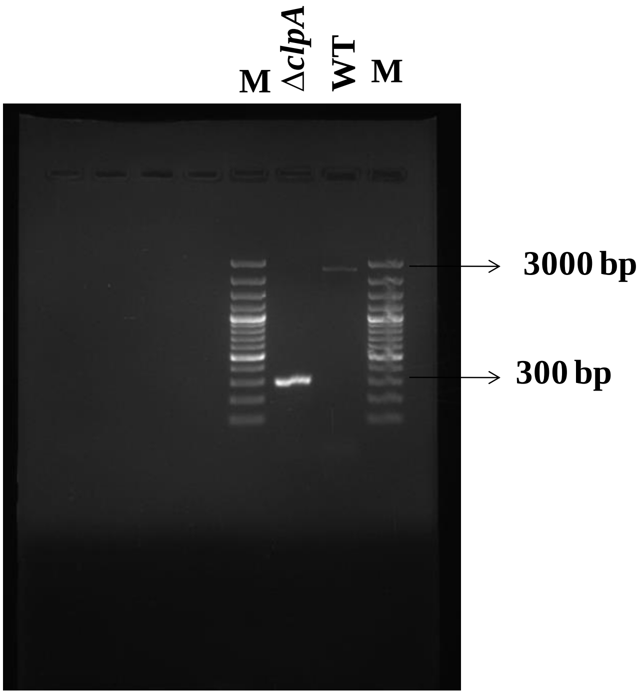

Full length gel of Supplementary Fig. S1 (B).

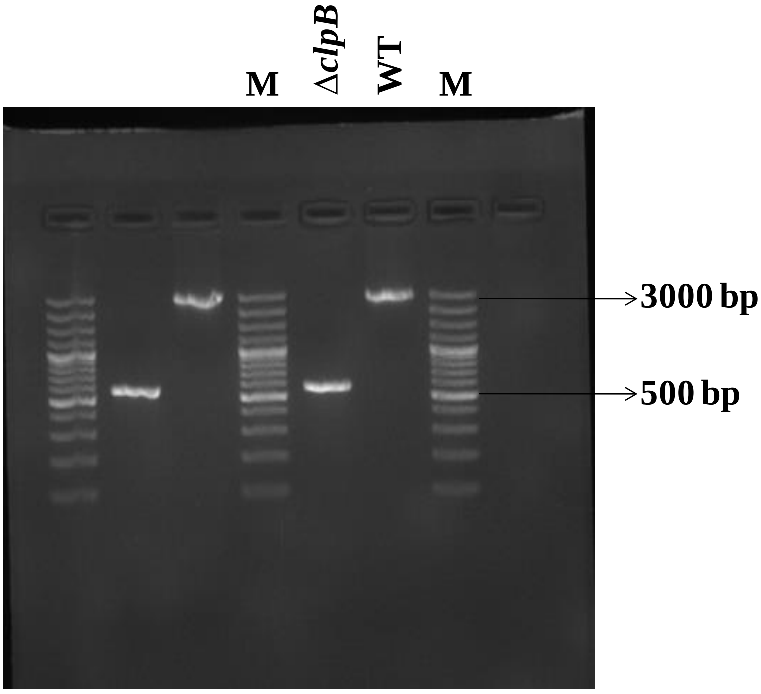

Supplement: Supplementary file 1 — Supplementary information [file 41598_2018_22670_MOESM1_ESM.pdf]
